# Retraction: Hydrogen sulfate ion sensing in aqueous media based on a fused pyrimido benzothiazole derivative

**DOI:** 10.1039/d3ra90003g

**Published:** 2023-01-13

**Authors:** Sachin D. Padghan, Rajesh S. Bhosale, Namdev V. Ghule, Avinash L. Puyad, Sheshanath V. Bhosale, Sidhanath V. Bhosale

**Affiliations:** a Polymers and Functional Materials Division, CSIR-Indian Institute of Chemical Technology Hyderabad-500007 Telangana India bhosale@iict.res.in; b School of Chemical Sciences, Swami Ramanand Teerth Marathwada University Nanded-431606 Maharashtra India; c School of Applied Sciences, RMIT University GPO Box 2476 Melbourne VIC-3001 Australia Sheshanath.bhosale@rmit.edu.au

## Abstract

Retraction of ‘Hydrogen sulfate ion sensing in aqueous media based on a fused pyrimido benzothiazole derivative’ by Sachin D. Padghan *et al.*, *RSC Adv.*, 2016, **6**, 34376–34380, https://doi.org/10.1039/C6RA01980C.

The Royal Society of Chemistry, with the agreement of the authors, hereby wholly retracts this *RSC Advances* article following an institutional investigation carried out by RMIT University.

Fig. 1 contains several identical vials representing different experiments.

The investigation found that this paper “breached the Australian Code and RMIT Research Policy by not ensuring that conclusions are justified by the results and not responsibly disseminating research findings”. A recommendation was given that this paper is retracted.

Dr Sachin D. Padghan was fully responsible for the error that happened in the figure. All other authors Rajesh S. Bhosale, Namdev V. Ghule, Avinash L. Puyad, Sheshanath V. Bhosale and Sidhanath V. Bhosale are not responsible for this error.

Signed: Sachin D. Padghan, Rajesh S. Bhosale, Namdev V. Ghule, Avinash L. Puyad, Sheshanath V. Bhosale and Sidhanath V. Bhosale

Date: 09/01/2023

## Supplementary Material

